# Persistent Clones and Local Seed Recruitment Contribute to the Resilience of *Enhalus acoroides* Populations Under Disturbance

**DOI:** 10.3389/fpls.2021.658213

**Published:** 2021-06-04

**Authors:** Jasper Dierick, Thi Thuy Hang Phan, Quang Doc Luong, Ludwig Triest

**Affiliations:** ^1^Ecology and Biodiversity Research Group, Biology Department, Vrije Universiteit Brussel (VUB), Brussels, Belgium; ^2^Biology Department, University of Sciences, Hue University, Hue, Vietnam

**Keywords:** *Enhalus*, disturbance, persistence, resilience, clonal richness, genetic diversity, microsatellites (SSR markers)

## Abstract

Human-induced land use in coastal areas is one of the main threats for seagrass meadows globally causing eutrophication and sedimentation. These environmental stressors induce sudden ecosystem shifts toward new alternative stable states defined by lower seagrass richness and abundance. *Enhalus acoroides*, a large-sized tropical seagrass species, appears to be more resistant toward environmental change compared to coexisting seagrass species. We hypothesize that reproductive strategy and the extent of seedling recruitment of *E. acoroides* are altered under disturbance and contribute to the persistence and resilience of *E. acoroides* meadows. In this research, we studied eight populations of *E. acoroides* in four lagoons along the South Central Coast of Vietnam using 11 polymorphic microsatellite loci. We classified land use in 6 classes based on Sentinel-2 L2A images and determined the effect of human-induced land use at different spatial scales on clonal richness and structure, fine-scale genetic structure and genetic diversity. No evidence of population size reductions due to disturbance was found, however, lagoons were strongly differentiated and may act as barriers to gene flow. The proportion and size of clones were significantly higher in populations of surrounding catchments with larger areas of agriculture, urbanization and aquaculture. We postulate that large resistant genets contribute to the resilience of *E. acoroides* meadows under high levels of disturbance. Although the importance of clonal growth increases with disturbance, sexual reproduction and the subsequent recruitment of seedlings remains an essential strategy for the persistence of populations of *E. acoroides* and should be prioritized in conservation measures to ensure broad-scale and long-term resilience toward future environmental change.

## Introduction

Seagrasses are marine aquatic angiosperms that can form dense meadows in shallow coastal waters. Two reproductive strategies exist where new genetic individuals are recruited as a result of sexual reproduction and once settled can reproduce clonally by rhizome extension with the vertical formation of genetically identical shoots (Kendrick et al., 2012, 2017; McMahon et al., 2014). Both modes act together and their relative contribution can be inferred from population genetic studies with the assessment of clonal diversity and structure (Arnaud-Haond et al., 2007). The level of clonality differs between species due to species-specific sexual (e.g., pollen, flower, fruit, and seed characteristics) and asexual traits (e.g., rhizome growth rate). Consequently, population growth and dispersal strategies vary between species with repeated recruitment of newly developed seeds as one extreme and one initial seed recruitment followed by vegetative growth as the other (Eriksson, 1993; Kendrick et al., 2012; McMahon et al., 2014). Temporal and spatial intraspecific variation in clonality and reproductive output has been described in seagrasses with disturbance, latitude, sea surface temperature and local scale processes as possible environmental drivers (Diaz-Almela et al., 2007; van Dijk and van Tussenbroek, 2010; McMahon et al., 2017; Connolly et al., 2018; Yue et al., 2020).

Seagrass ecosystems are threatened globally with ocean warming (Duarte et al., 2018; Kendrick et al., 2019), coastal development and related human activities (Waycott et al., 2009; Grech et al., 2011; Unsworth et al., 2019) recognized as the most important drivers of seagrass decline. Deforestation and runoff from urban, agricultural and industrial areas result in sedimentation and eutrophication which imposes direct threats to seagrass meadows due to diminished light conditions, sedimental burial and indirect feedback effects (e.g., sediment anoxia and re-suspension, trophic alterations) (Terrados et al., 1998; Waycott et al., 2009; Quiros et al., 2017). Depending on the strength and frequency of environmental perturbations, disturbance can induce sudden shifts to new alternative stable states characterized by low seagrass richness and abundance and increased growth of macro-algae, epiphytic algae and phytoplankton (Burkholder et al., 2007; Waycott et al., 2009; Quiros et al., 2017). The resilience of seagrass communities toward disturbances is largely determined by species richness and composition with fast-growing species relying on recovery by seed banks and clonal growth whereas climax species generally have a higher resistance toward stressors due to carbohydrate and nutrient storage in rhizomes and rely mainly on local and regional recruitment of seedlings (Marbà et al., 1996; Walker et al., 1999; Olesen et al., 2004; Kendrick et al., 2012, 2019; Unsworth et al., 2015; Viana et al., 2020). Clonal growth and seedling recruitment are therefore contrasting strategies for the maintenance and persistence of populations under disturbance and affect genotypic richness, genetic richness and genetic connectivity; hence these strategies are important components for population resilience (Unsworth et al., 2015). Contrasting patterns described positive, negative or no relationships between disturbance, clonality and sexual output depending on study species, type, strength and frequency of disturbance (Reusch, 2006; Diaz-Almela et al., 2007; van Dijk and van Tussenbroek, 2010; McMahon et al., 2017; Phan et al., 2017; Connolly et al., 2018).

*Enhalus acoroides* is a tropical, large-sized, dioecious seagrass species whose female flowers are connected with long peduncles and are pollinated by free-floating, detached male flowers on the water surface (epihydrophily) (Les et al., 1997; Tanaka et al., 2004). Long-distance export outside natural *Enhalus* stands may be considerable since both mature fruits (when dislodged) and seeds have the capacity to float for days and hours, respectively (Lacap et al., 2002; Rollón et al., 2003). Fruits can disperse up to 63.5 km (Lacap et al., 2002) and contain 8–14 non-dormant seeds which are released in the water column (Ackerman, 2006), hence *E. acoroides* does not rely on seed banks for recruitment. Sexual reproduction appears to be more important for the maintenance and persistence of *E. acoroides* populations compared to clonal growth (Rollón et al., 2003; McMahon et al., 2017; Yu et al., 2018, 2019; Nguyen and Jutta, 2019) although a variety of sampling methods (e.g., spacious distance between sampled shoots) could underestimate the role of asexual reproduction. Quiros et al. (2017) studied the effect of human-induced disturbance on seagrass communities and showed that sites characterized by large areas of coastal human development and farmland had lower species richness but increased *E. acoroides* cover. Other studies confirm the high resistance of *E. acoroides* toward eutrophication and sedimentation (Duarte et al., 1997; Terrados et al., 1998; Kiswara et al., 2005; Quiros et al., 2017) which may be the result of their large leaves enabling photosynthesis in turbid water and the presence of nutrient reserves in rhizomes and seeds (Marbà et al., 1996; Artika et al., 2020).

Our goal was to investigate the mechanisms for population maintenance of *E. acoroides* under disturbance to understand how meadows of *E. acoroides* persist in human-altered environments. More specifically, we aimed to assess (1) the response of populations due to potential size reductions, (2) the importance of local within-lagoon recruitment and regional among-lagoon connectivity and (3) the contribution of sexual and asexual reproduction under disturbance. Therefore, we determined clonal richness and structure, genetic diversity and levels of dispersal within and between eight populations of *E. acoroides* in four lagoons along the South Central Coast of Vietnam and assessed the relationship between genetic variables and human-induced land use over spatial scales. We hypothesize that disturbance characterized by large areas of human-induced land use affects the genetic diversity, reproductive strategy and the extent of seedling recruitment in populations of *E. acoroides.*

## Materials and Methods

### Study Area

**FIGURE 1:**
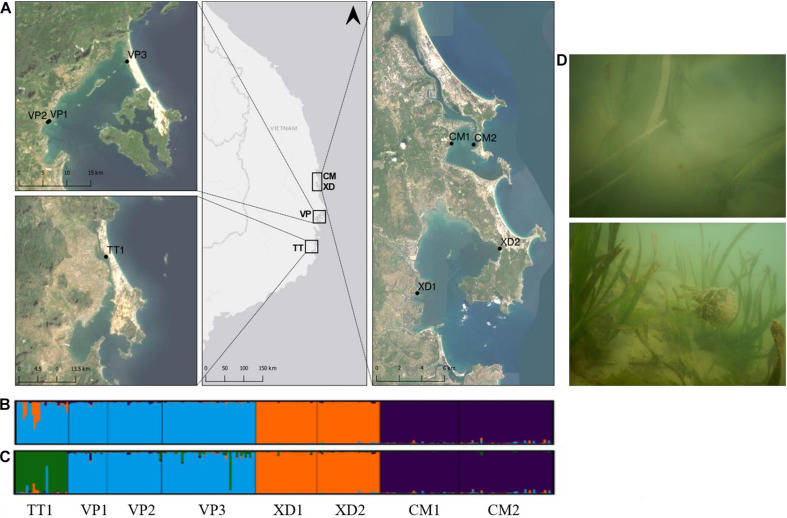
**(A)** Sampled *Enhalus acoroides* populations along the South Central Coast of Vietnam in four lagoons; Thuy Trieu (TT), Van Phong Bay (VP), Xuan day Bay (XD), Cu Mong (CM). Map generated using the Quantum Geographical Information System, version 3.10.3 (QGIS). Basemap source: ArcGIS. **(B,C)** Results of Bayesian clustering analysis (STRUCTURE v.2.3.4) generated by Structure Harvester with the number of groups for **(B)** being K = 3, **(C)** being K = 4. **(D)** Photographs of sampled *E. acoroides* meadows in Van Phong Bay (above) and Xuan Day Bay (below).

The study area was located in the provinces Khanh Hoa (Thuy Trieu and Van Phong Bay) and Phu Yen (Xuan Day and Cu Mong) (Figure 1A). Studies determined seagrass distribution over time and identified possible drivers for seagrass loss in Khanh Hoa which revealed a total loss of 74.2% of seagrass area between 2008 and 2018 and various human activities in the coastal zone (e.g., tourism, shellfish collection and aquaculture) appeared as main causes of seagrass decline (Chen et al., 2016; Khanh Ni et al., 2020). In Van Phong Bay, a total decrease of 35.8% over 30 years (1988–2019) with an approximate loss of 46.5–87.3 Ha per decade has been shown (Vo et al., 2020). Consequently, seagrass meadows in Thuy Thrieu and Van Phong Bay are fragmented and restricted to shallow waters with a maximum depth of 3.5–5 m near the coast (Khanh Ni et al., 2020; Vo et al., 2020). Thieu Thrieu is characterized by high turbidity with spatio-temporal variations driven by rainfall and resuspension of the sediment with maximum values for NTU exceeding 13.1 (Quang et al., 2017). Additionally, studies confirmed enrichments of Cu and other metals (As, Cr, Ni, Pb, Zn) due to shipping activities in Khanh Hoa ranging from moderate to severe (Dung et al., 2014) which could affect seagrass beds (Nguyen et al., 2017). The rapid expansion of aquaculture area (especially lobster farming) in recent years has caused eutrophication along the coast of Phu Yen (Hoang et al., 2018), however, no information on seagrass distribution and change is currently available for this province.

### Sampling Collection

A total of 384 individual shoots of *E. acoroides* were collected in April 2019 at 8 populations in four different lagoons along the South Central Coast of Vietnam (Figure 1A and Table 1). We sampled along transects with an interval of 1 meter and collected approximately 50 individual shoots for each site. This sampling method ensures an adequate resolution to determine clonal characteristics and fine-scale structure within populations and should provide sufficient unique multilocus genotypes (MLG’s). Samples were dried in envelopes and brought to Vrije Universiteit Brussel (Belgium) for further analysis. GPS coordinates at the starting point of each transect were taken. Sampling depth varied between 50 and 140 cm. Turbidity, salinity, pH and temperature were measured (Horiba U-50 Series multi-parameter) in all sites and ranged from 2.5 NTU to 17.5 NTU, 34.1 ppt to 37.2 ppt, 8.62 to 9.02, and 31.3 to 33.75°C. The surface layer of sediment consisted of fine sand and mud. High abundances of algae and Cnidaria (jellyfish and anemones) were present in the water and on seagrass shoots. All sampled meadows were monospecific except for VP3 where the seagrass species *Thalassia hemprichii* was found intermingled with *E. acoroides* and TT1 where a monospecific patch of *Halophila ovalis* existed at 2 m distance from the sampled transect.

**TABLE 1:** Details of eight *Enhalus acoroides* populations along the South Central Coast of Vietnam with catchment area and absolute and relative areas for each land use factor within a 10 km radius of sampling sites.

Site	Lagoon	Lat (N)	Long (E)	Catchment	Forest		Agriculture		Urbanization		Aquaculture		Bare soil		Unvegetated	
				km^2^	km^2^	%	km^2^	%	km^2^	%	km^2^	%	km^2^	%	km^2^	%
TT1	Thuy Trieu	12.043	109.196	166.01	16.64	0.10	60.22	0.36	27.14	0.16	11.02	0.07	10.83	0.07	40.15	0.24
VP1	Van Phong Bay	12.648	109.206	146.27	31.39	0.21	61.27	0.42	12.46	0.09	12.73	0.09	0.81	0.01	27.61	0.19
VP2	Van Phong Bay	12.646	109.203	145.38	31.28	0.22	60.27	0.41	12.63	0.09	12.71	0.09	0.85	0.01	27.64	0.19
VP3	Van Phong Bay	12.758	109.355	82.32	31.50	0.38	23.91	0.29	4.61	0.06	8.49	0.10	5.63	0.07	8.19	0.10
XD1	Xuan Day Bay	13.421	109.225	148.05	67.17	0.45	41.06	0.28	5.91	0.04	3.45	0.02	0.73	0.00	29.73	0.20
XD2	Xuan Day Bay	13.456	109.293	60.87	25.60	0.42	16.66	0.27	3.62	0.06	2.09	0.03	0.45	0.01	12.44	0.20
CM1	Cu Mong	13.539	109.253	71.27	30.73	0.43	18.11	0.25	2.97	0.04	5.18	0.07	0.72	0.01	13.57	0.19
CM2	Cu Mong	13.538	109.271	60.84	25.37	0.42	15.47	0.25	2.89	0.05	4.79	0.08	0.71	0.01	11.61	0.19

### DNA Extraction and Microsatellite Primers

Genomic DNA was extracted from approximately 20 mg of dried leaf tissue using the E.Z.N.A. SP plant DNA Mini kit (Omega bio-tek, Norcross, GA, United States). Eleven polymorphic microsatellite markers (EA447, EA1461, Eaco_001, Eaco_002, Eaco_009, Eaco_048, Eaco_050, Eaco_051, Eaco_052, Eaco_054, Eaco_055) developed for *E. acoroides* (Gao et al., 2012; Nakajima et al., 2012) were selected for genotyping (Supplementary Table 1). Primers were fluorescence-labeled with 4 different dye-labels (6FAM/VIC/NED/PET) and a primer mix was made by mixing 0.2 μM of each primer. Multiplex PCR reactions consisted of 6.25 μl master mix (Qiagen Multiplex PCR kit), 1.25 μl primer mix, 2.5 μl H_2_O and 2.5 μl of genomic DNA. PCR was performed in a thermal cycler (Bio-Rad MyCycler) with the following conditions: an initial denaturation of 95°C for 15 min followed by 35 cycles of: 30 s denaturation at 95°C, 90 s annealing at 57°C and 80 s elongation at 72°C followed by a final extension of 30 min at 60°C. PCR products were separated on an ABI3730XL sequencer (Macrogen, Seoul, Korea) and allele sizes were determined with GeneMarker (v.2.60) (Holland and Parson, 2011).

### Genetic Analyses

#### Null-Alleles, Scoring Errors, Linkage Disequilibrium, and Probability of Identity

From 384 collected shoots, 367 gave fully interpretable PCR products. Preceding population genetic analyses, we tested for the presence of potential null alleles, scoring errors and linkage disequilibrium. No scoring errors or null alleles were found using MICRO-CHECKER (Van Oosterhout et al., 2004). A linkage test between all pairs of loci (1,000 permutations) after the removal of shared MLG’s detected no genotypic disequilibrium at the significance level of 0.05 for the combination of all loci using FSTAT (v.2.9.3) (Goudet, 2001). The overall resolution of the eleven used microsatellites was verified by calculating the probability of identity (PI) using GenAlEx (v.6.6) (Peakall and Smouse, 2012) and gave a cumulative PI smaller than 0.01 for all populations.

#### Clonal Diversity and Structure

To confirm clonal identity between individuals with identical MLG’s we calculated the probability that identical MLG’s have arisen from distinct sexual reproduction events (p_sex_) (Parks and Werth, 1993; Arnaud-Haond et al., 2007). For three replicates of a shared MLG in XD1 we found a p_sex_ value larger than 0.05; consequently, we did not consider these cases as clones of the same genet. Additionally, we screened genotype accumulation curves and genetic distance histograms for each site as proposed in Arnaud-Haond et al. (2007) and did not find any discrepancies which could lead to an underestimation of the proportion of shared MLG’s and thus clones.

Using RClone (Bailleul et al., 2016) we calculated three indices to describe clonal diversity which are least redundant in comparison with other diversity indices (Arnaud-Haond et al., 2007), namely clonal richness (R), which is the proportion of genets (G) on the total number of sampling units or ramets (N) (Dorken and Eckert, 2001); the Simpson’s evenness index (V) and the Pareto index (β). To assess segregation patterns of clones we determined the aggregation index (A_c_), the probability that nearest neighbors are clones of the same genet relative to randomly drawn pairs (Arnaud-Haond et al., 2007), and was significantly tested with 1,000 permutations. Additionally, we calculated the maximum number of ramets per genet for each site (N_Gmax_) together with clonal subrange (CS), the maximum distance interval at which the chance of clonal identity exceeds 0.

#### Fine-Scale Genetic Structure

Fine-scale spatial autocorrelation analyses were performed to assess the relationship between kinship coefficient (F_ij_) (Loiselle et al., 1995) and spatial distance between pairs of individuals at transect level using SPAGeDi (v.1.5a) (Hardy and Vekemans, 2002). Kinship coefficients were calculated over eight distance classes (0–1, 1–2, 2–3, 3–5, 5–10, 10–20, 20–30, 30–50 m) and significantly tested, using within-category as reference, together with the slope (b) of the regression with 1,000 permutations. To determine both the spatial field over which clonality affects the genetic structure as the range of seed and pollen dispersal within a population, two analyses per transect were performed: (1) among ramets analysis: kinship analysis between all pairs including ramets of the same genet; (2) among genets analysis: kinship analysis between pairs of ramets of different genets (Supplementary Figure 1). For both analyses the Sp-statistic was calculated as Sp = −blog/(1−F1) (Vekemans and Hardy, 2004), where b_log_ is the slope of the ln regression and F_1_ the average kinship coefficient between individuals in the first distance class (0–1 m) (Supplementary Table 2).

#### Genetic Diversity and Population Structure

Genetic diversity and population structure analyses were performed on a dataset including one individual for each genet by removing all individuals which were identified as clones (see section “Clonal Diversity and Structure”) except one. Effective number of alleles (N_e_), allelic richness (AR) for 16 diploid samples, observed heterozygosity (H_obs_), expected heterozygosity (H_exp_) and population inbreeding coefficient (F_is_) were calculated for each site using GenAlEx and FSTAT.

We carried out a hierarchical three-level Analysis of Molecular Variance (AMOVA) and calculated F-statistics with 999 random permutations (Michalakis and Excoffier, 1996) to study genetic structure among regions (with lagoons considered as regions), among populations, among individuals and within individuals. Additionally, the pairwise F_st_ matrix between populations was calculated and tested with 999 random permutations (Supplementary Table 3). A Bayesian clustering analysis was performed in STRUCTURE (v.2.3.4) (Pritchard et al., 2000) to assign individuals to populations using an admixture model (10 iterations for each K; 50,000 burn-in period; 500,000 Markov chain Monte Carlo repeats). Within StructureSelector (Li and Liu, 2018), Structure Harvester (Earl and vonHoldt, 2012) and CLUMPAK (Kopelman et al., 2015) were used to determine the optimal number of clusters (k) (Evanno et al., 2005; Supplementary Figure 2) and graphically present the STRUCTURE results.

### Satellite Imagery and Land Use Classification

We suggest that the area of human-induced land use is an indirect but integrative proxy for human-induced disturbance especially in habitats (i.e., lagoons) characterized by high environmental fluctuations (water depth, turbidity, salinity). Therefore, we quantified land use in lagoons using remote sensing data. Satellite imagery was achieved by Sentinel-2 and downloaded from ESA Copernicus Open Access Hub^1^. We targeted Sentinel-2 L2A images with atmospheric correction sensed between February and June 2019 with a maximum cloud cover of 5%. Twelve spectral bands were resampled to a pixel size of 10m, merged and exported as raster files using Sentinel Toolbox SNAP (v.2.0.2) (ESA Sentinel Application Platform). Satellite raster files were merged with the software QGIS (v.3.10.3) (QGIS org, 2021) and consequently, we obtained one raster file with spectral data covering all four lagoons. Based on the satellite-2 image we identified training samples and classified six land use classes (forest, urbanization, agriculture, bare soil, unvegetated area, water) with a random forest classifier using EnMap-Box in QGIS (Supplementary Figure 3). We carried out an accuracy assessment analysis based on validation samples including total accuracy (90.48%), kappa accuracy (84.74%) and user’s and producer’s accuracy for each class accompanied with the confusion matrix (Supplementary Tables 4, 5). Additionally, we classified aquaculture manually as an additional land use class. Catchment area boundaries of the four sampled lagoons were determined using Digital Elevation Models in QGIS. For each lagoon, we calculated the catchment area excluding water and the absolute and relative area of land use for each class within the catchment. To assess the effect of land use at different spatial scales we calculated catchment and land use area around populations within spatial buffers of four different radiuses (0.5, 1, 5, and 10 km) and within the catchment area of the total lagoon (Supplementary Table 6).

### Effect of Land Use on Genetic Variables

**FIGURE 2:**
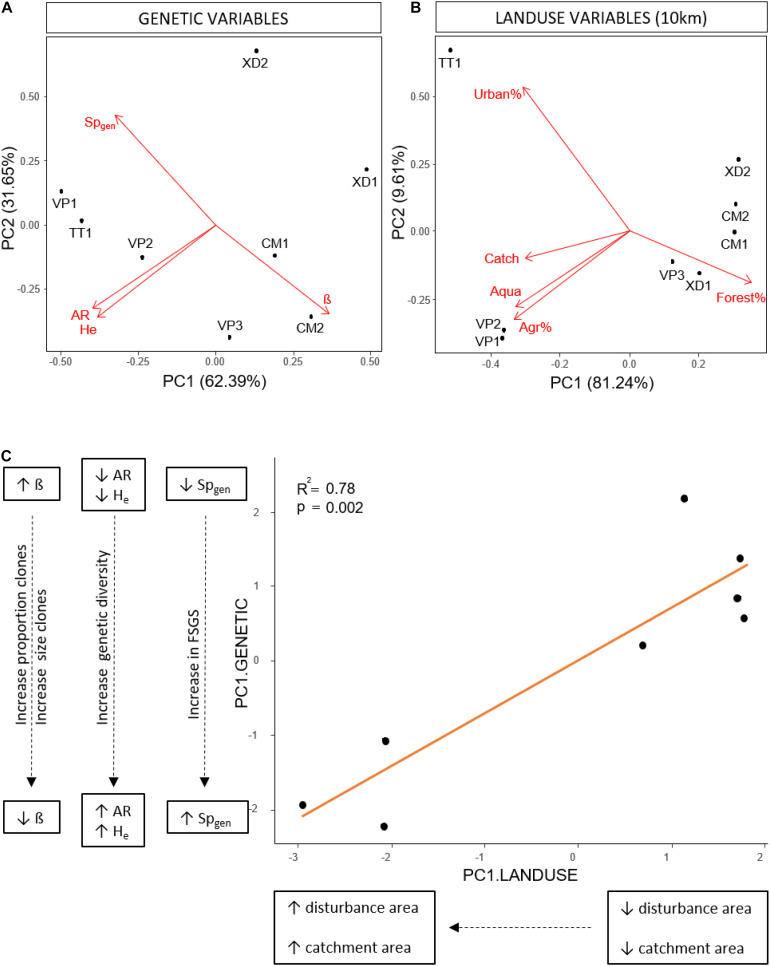
**(A)** Principal component analysis of population genetic variables; Pareto index (β), among genet Sp-statistic (Sp_gen_), effective number of alleles (N_e_), allelic richness (AR), expected heterozygosity (H_exp_). **(B)** Principal component analysis of land use variables on the 10 km spatial scale; absolute catchment area (Catch), relative area of forest (Forest%), relative area of agriculture (Agr%), relative area of urbanization (Urban%), absolute area of aquaculture (Aqua). **(C)** Linear regression between the transformed land use variables (PC1.LAND USE) and the transformed genetic variables (PC1.GENETIC) based on the first axis of the PCA’s in respectively **(B)** and **(A)**, showing an increase in the proportion and size of clones, genetic diversity and fine-scale genetic structure with increasing catchment and human-induced land use area.

We performed a Principal Component Analysis (PCA) on genetic parameters (AR, H_e_, β and Sp_gen_) using R (R Core Team, 2021). Populations were organized along the first axis (62,39%) with an increase in Pareto index (β), a decrease in genetic diversity (AR, He) and a decrease in fine-scale genetic structure (Sp_gen_) with increasing PC1 values (Figure 2A) and transformed the genetic variables based on the first axis of the genetic PCA (PC1.GENETIC). Based on the acquired land use data, we selected 5 factors that were highly consistent with human-induced land use (catchment, urban, agriculture, aquaculture and forest area) and tested their effect and the effect of spatial scale on the transformed genetic variables. Because the area of urbanization, agriculture and forest was highly correlated with the catchment area we used the relative areas of these land use factors in the analyses to decouple the effect of catchment area from land use. We determined the effect for each land use class separately by linear regressions over different spatial scales and corrected for multiple tests within each scale (Holm-Bonferroni method). The strongest relationship between human-induced land use and the transformed genetic variables was found on the 10 km spatial scale; consequently, we performed an additional PCA on land use variables on this scale. Two groups of populations along the first axis (81.24%) were identified with TT1, VP1 and VP2 characterized by large catchment sizes, large areas of aquaculture, agriculture and urbanization, and low areas of forest (Figure 2B). We transformed land use variables based on the first axis (PC1.LANDUSE) and performed an additional linear regression between the transformed variables of both PCA’s (PC1.GENETIC, PC1.LANDUSE) to test the overall effect of land use factors within a radius of 10km on clonal diversity, genetic diversity and fine-scale genetic structure.

## Results

### Clonal Diversity and Structure

A total of 247 unique multilocus genotypes were identified for 367 *E. acoroides* samples. Clonal diversity differed between populations with large variation in clonal richness (*R* = 0.67) ranging from 0.36 to 0.89 and Pareto index (β = 1.26) from 0.47 to 2.26 (Table 2). Clonal evenness (V = 0.86) was overall high and similar between populations indicating that lineages of multilocus genotypes are present in more or less equal abundances (Table 2). The maximum number of ramets per genet (N_Gmax_ = 4.5) and clonal subrange (CS = 5.9 m) varied with a range from 2 to 8 and 1 to 13 m, respectively (Table 2). Remarkably, XD2 which has the second-largest clonal subrange only has a small number of maximum ramets per genet which is explained by one clone harboring a space up to 11 m but which only consists of four ramets and could be an indication of small scale dispersal by clonal fragments or fragmentation of ramets. β was strongly correlated with R (*R*^2^ = 0.94), negatively correlated with N_Gmax_ (*R*^2^ = –0.73) but not significantly correlated with V (*R*^2^ = –0.65) showing that populations with a low Pareto index have higher proportions of clones and contain larger genets. For all populations, the aggregation coefficient (A_C_) was significant and ranged from 0.47 to 0.77 which infers high spatial aggregation of ramets (Table 2).

**TABLE 2:** Population genetic variables for eight populations of *Enhalus acoroides* along the South Central Coast of Vietnam.

Site	Clonal diversity and structure								FSGS		Genetic diversity					
	N	G	R	V	β	A_c_	N_Gmax_	CS	Sp_ram_	Sp_gen_	N_a_	N_e_	AR	H_obs_	H_exp_	F_is_
TT1	46	24	0.51	0.87	0.86**	0.58***	8	13	0.151	0.097	3.55	2.09	3.33	0.431	0.410	–0.028
VP1	48	18	0.36	0.88	0.47*	0.77***	8	7	0.211	0.098	3.55	2.35	3.48	0.391	0.389	0.025
VP2	49	25	0.50	0.94	0.77*	0.60***	4	7	0.070	0.030	3.64	2.10	3.30	0.409	0.372	–0.077
VP3	52	43	0.82	0.78	1.74*	0.52***	4	3	0.014	0.000	3.64	2.05	3.20	0.369	0.363	–0.004
XD1	37	29	0.78	0.81	1.6*	0.49***	4	3	0.024	0.006	2.09	1.40	2.00	0.239	0.225	–0.043
XD2	43	29	0.67	0.86	0.93**	0.54***	4	11	0.119	0.096	2.00	1.51	1.95	0.263	0.254	–0.017
CM1	44	36	0.81	0.90	1.46	0.54***	2	1	0.013	0.001	3.00	1.71	2.55	0.360	0.321	–0.105
CM2	48	43	0.89	0.82	2.26	0.47***	2	2	0.007	0.002	2.91	1.79	2.52	0.343	0.342	0.010
Total or mean	367	247	0.67	0.86	1.26	0.56	4.5	5.9	0.068	0.038	3.05	1.88	2.79	0.351	0.335	–0.030

N, Total number of samples/ramets; G, total number of genets; R, clonal richness; V, Simpson’s evenness index; β, Pareto index; A_ c_, aggregation index; N_ Gmax_, the maximum number of ramets per genet; CS, clonal subrange; Sp_ ram_, among ramet Sp-statistic; Sp_ gen_, among genet Sp-statistic; N_ a,_ mean number of alleles; N_ e,_ effective number of alleles; AR, allelic richness for 16 diploid samples; H_ obs,_ observed heterozygosity; H_ exp,_ expected heterozygosity; F_ is,_ inbreeding coefficient. Significance levels are indicated as follows (only applicable for β and A_ c_): ***significant at p < 0.001, **significant at p < 0.01, *significant at p < 0.05.

### Fine-Scale Genetic Structure

The among ramet spatial autocorrelation analysis gave a significant mean kinship coefficient for the first distance class (F_i__1r__am_ = 0.190) for all populations with the logarithmic regression slope (b) being significant over 50 m except for CM2 (Supplementary Table 2). The among-ramet *Sp*-statistic (Sp_ram_) varied greatly between populations from 0.007 to 0.211 (Table 2). Similar results were obtained for the among genets analysis with lower but still significant kinship values (F_i__1r__am_ = 0.091) for all populations except VP3, significant slopes for TT1,VP1,VP2 and XD1 ((Supplementary Table 2) and among-genet Sp-statistics (Sp_gen_ = 0.041) ranging from 0 to 0.098 (Table 2). Sp_gen_ was negatively correlated with R (*R* = –0.78) and positively with N_Gmax_ (*R* = 0.89) indicating that clonal structure affects the fine-scale structure of populations even when clone pairs are not considered. TT1, VP1, VP2, and XD2 have a higher fine-scale genetic structure for both analyses with a maximum distance ranging from 5 to 10 m depending on site and analysis (Supplementary Figure 1).

### Genetic Diversity and Population Structure

Number of alleles (N_a_ = 3.05), effective number of alleles, N_e_ = 1.88) and allelic richness (AR = 2.79) varied between populations and ranged from 2.09 to 3.64, 1.40 to 2.35 and 1.95 to 3.48, respectively (Table 2). Values for expected and observed heterozygosity were similar within each population and the within-population inbreeding (F_is_ = −0.030) was close to zero and nowhere significant (Table 2). AR and H_e_ were strongly correlated (*R*^2^ = 0.94) indicating that those alleles that contribute to a higher allelic richness in populations are generally found in a heterozygous state.

The three-level AMOVA analysis (Table 3) showed that a high proportion of the molecular variance was explained among regions (33%) but only a small proportion among populations within lagoons (5%) with F_rt_ and F_sr_ values being, respectively, 0.327 and 0.081. These results, together with high Fst *P*-values of populations between and low Fst *P*-values within lagoons (Supplementary Table 3), indicate high differentiation between populations among lagoons but low differentiation and thus high genetic connectivity of populations within lagoons. The highest Fst *P*-value (0.518) was found between XD1 and CM1 which could indicate a barrier between neighboring lagoons XD and CM (Supplementary Table 3). The AMOVA inbreeding coefficient (F_is_ = 0.044) was low with a small proportion of the variation explained by individuals within populations (3%). In contrast, the largest proportion was found within individuals (59%) (Table 3). Bayesian clustering analysis revealed *k* = 3 (Delta *K* = 1371) as the most likely number of gene pools (Supplementary Figure 2) with individuals clustered almost exclusively to their respective lagoon (blue = VP lagoon, orange = XD lagoon, purple = CM lagoon) (Figure 1B) except for TT1 where most individuals were fully assigned to the VP cluster and a small proportion was admixed between the VP and XD cluster. With *k* = 4, the largest proportion (92%) of individuals of TT1 were grouped together in an additional cluster (green) (Figure 1C) and verifies the AMOVA results which indicate high connectivity within but low connectivity between lagoons.

**TABLE 3:** Summary of hierarchical three-level AMOVA and F-statistics of *Enhalus acoroides* for eight populations along the South Central Coast of Vietnam; df, degrees of freedom; SS, sum of squares; MS, mean of squares; % Est.Var., estimated variance.

Source	df	SS	MS	Est. Var.	%	F-statistics	*p*-value
Among regions	3	397.630	132.543	1.029	33%	F_rt_ = 0.327	0.001
Among Pops	4	49.682	12.421	0.172	5%	F_sr_ = 0.081	0.001
Among Indiv	238	482.753	2.028	0.086	3%	F_st_ = 0.382	0.001
Within Indiv	246	456.500	1.856	1.856	59%	F_is_ = 0.044	0.008
						F_it_ = 0.409	0.001

### Effect of Land Use on Genetic Variables

Variation in land use variables was found with a consistent pattern on large spatial scales (10 km, total lagoon) with TT1, VP1, VP2 present in areas with larger catchment sizes and higher levels of disturbance characterized by larger areas of urbanization, agriculture and aquaculture compared to VP3, XD1, XD2, CM1, and CM2 (Table 1 and Supplementary Table 6). We identified no similar and uniform pattern at smaller scales (0.5, 1, 5 km) where differences in land use area between populations were scale-dependent (Supplementary Table 6). We found no significant effect of human-induced land use area on the measured environmental parameters (turbidity, salinity, pH, and temperature). Linear regression analyses per land use factor on PC1.GENETIC showed significant negative effects of aquaculture, agriculture and urbanization on large scales (10 km, lagoon) and of catchment size for the total lagoon (Table 4). No significant relationship was found on smaller spatial scales (0.5, 1, 5 km) (Table 4) showing that human-induced land use only has an effect on clonal diversity, genetic diversity and fine-scale genetic structure of populations on large spatial scales. A positive significant relationship between PC1.LANDUSE and PC1.GENETIC (*p* = 0.002) revealed a decrease in Pareto index (β), an increase in genetic diversity (AR, H_e_) and an increase in fine-scale genetic structure (Sp_gen_) in populations inhabiting areas with larger human-induced land use areas, larger catchments areas and smaller forest areas on the 10 km spatial scale (Figure 2C).

**TABLE 4:** Summary of linear regressions between the transformed genetic variables based on the first axis of the PCA and land use factors for five spatial scales (0.5, 1, 5, 10 km, lagoon).

Buffer	Aquaculture (km^2^)			Agriculture %			Urbanization %			Catchment (km^2^)		
	*R*^2^_Adj_	*b*	*p*-value	*R*^2^_Adj_	*b*	*p*-value	*R*^2^_Adj_	*b*	*p*-value	*R*^2^_Adj_	*b*	*p*-value
0.5 km	−0.11	8.56	ns	−0.09	3.34	ns	0.18	−2.99	ns	−0.14	2.31	ns
1 km	0.04	−5.92	ns	−0.13	2.33	ns	0.19	−4.68	ns	−0.16	−0.47	ns
5 km	0.47	−0.71	ns	0.03	−6.54	ns	0.45	−19.39	ns	−0.09	−0.04	ns
10 km	0.74	−0.33	0.004	0.72	−19.80	0.005	0.58	−30.80	0.018	0.20	−0.02	ns
Lagoon	0.68	−0.14	0.007	0.59	−21.32	0.016	0.58	−59.43	0.017	0.55	−0.01	0.021

*The significance of p-values is corrected for multiple tests within each scale (Holm-Bonferroni method) and ns: not significant.*

## Discussion

### Genetic Diversity and Resilience of *E. acoroides* Populations Under Disturbance

Human activities consisting of agriculture, urbanization and aquaculture were broadly present in the sampling area though the area of human-induced land use differed between lagoons and over spatial scales. Water transparency was low at sites with high abundances of epiphytic algae, anemones and jellyfish in the water and on seagrass shoots (Figure 1D) which indicate that the sampled lagoons were highly eutrophic (Purcell et al., 2007); however, no relationship between land use and turbidity and no effect of turbidity on the genetic variables of *E. acoroides* populations could be found. Nevertheless, extreme reductions in seagrass coverage in our study area have been shown and are most likely associated with extensive human activities in lagoons and high levels of turbidity (Khanh Ni et al., 2020; Vo et al., 2020). No evidence of inbreeding or loss of genetic diversity was found for *E. acoroides* and demonstrates the high level of resilience of *E. acoroides* populations toward environmental perturbations. Still, it remains challenging to infer demographic responses directly from genetic diversity because no knowledge is available on the genetic diversity of populations before, during and after disturbances (Arnaud-Haond et al., 2010). The level of genetic diversity in our study is comparable to populations of Hainan Island, China (Yu et al., 2018, 2019), using a nearly similar set of loci, though lower compared to populations in the Philippines based on comparisons among analogous loci (Putra et al., 2018). Populations in Central Vietnam and South China are located on the margins of the species distribution range of *E. acoroides* (Short and Waycott, 2010) and lower levels of genetic diversity are most likely caused by range edge effects (Arnaud-Haond et al., 2006; Nakajima et al., 2014). A similar study on *E. acoroides* along the South Central Coast of Vietnam revealed higher levels of genetic diversity though nearly all sites were significantly inbred (Nguyen and Jutta, 2019). This presumably can be explained from a lower number (five) though highly polymorphic microsatellite markers used and their lower sample size (*N* = 10) which usually does not allow to detect a sufficient number of heterozygous genotypic combinations.

### Local Seed Recruitment Important for Population Resilience

Both AMOVA and STRUCTURE analyses show that lagoons are strongly differentiated and confirm the low levels of genetic connectivity found between populations in lagoons with the open sea (Nguyen and Jutta, 2019). Lagoons are isolated by land barriers with often small inlets and thus may act as barriers to gene flow. In contrast, low differentiation and the absence of genetic structure among populations within lagoons indicate that local recruitment by seedlings, originated within the meadow or within sites in the same lagoon, contributes greatly to the maintenance of *E. acoroides* populations. Olesen et al. (2004) studied the recovery of available spaces after disturbance in seagrass meadows and showed that sexual reproduction is highly important for the colonization of empty gaps. Direct mortality due to disturbance and the subsequent recruitment of new seedlings in available spaces from various sources within the lagoon could therefore positively affect genetic diversity which may explain the increased levels of allelic richness and heterozygosity under high levels of disturbance in our study.

A higher relatedness of individuals over small distances within meadows (5–10 m) was found in more disturbed sites on both ramet level, due to the aggregation of clones over several meters, and genet level. The aggregated distribution of pollen in male floating flowers and of seeds that are released simultaneously from the floating fruit could explain the fine-scale genetic structure found for *E. acoroides* among genets rather than pollen and seed dispersal restriction (Yu et al., 2019). The effect of the aggregated dispersal of propagules on the fine-scale structure may be elevated in disturbed sites when population density is low as a result of high mortality and decreased reproductive output. The successful settling and germination of seeds from the same fruit which are resistant to environmental stressors may be an additional explanation for the genetic structure within disturbed meadows. The influence of clonal structure on the fine-scale genetic structure among genets is less clear and understudied. Possible mechanisms are successful mating between a few clonal lineages, flower synchronization among ramets (Jahnke et al., 2015) or the aggregation of clones with slightly different but related genotypes as a result of somatic mutations (Yu et al., 2020).

No shared multilocus genotypes were found among populations, even within the same lagoon, hence genetic connectivity between populations of *E. acoroides* is only realized by sexual propagules and confirms the assumption that successful dispersal of vegetative fragments of large-sized seagrass species is low to non-existent (Lai et al., 2018). The genetic structure among lagoons is not explained by geographic distance with distant lagoons Thieu Thrieu and Van Phong Bay (~70 km distance) being less differentiated than neighboring lagoons Xuan Day Bay and Cu Mong (~14 km distance). Populations among lagoons with high levels of disturbance (TT and VP) appear to be less differentiated in our study. Human mediated dispersal of fruits and seeds between areas with higher levels of human activity may increase genetic connectivity among disturbed sites (TT and VP) (Orth et al., 2006; Bullock et al., 2018) though no evidence of direct human-mediated transport of propagules is available for *E. acoroides*. Clonal growth contributes to the persistence of populations under disturbance.

Populations of *E. acoroides* in our study are sustained by clonal growth and local seed recruitment which confirms the mixed reproduction strategy in populations of Hainan Island affected by physical disturbance and catchment effluents (Yu et al., 2019). Becheler et al. (2014) studied temporal dynamics of clonal structure for *Zostera marina* and identified similar patterns of clonal richness and structure whereas populations persisted by both initial recruitment, with a stable core of clones which dominance increased over time, and repeated (annual) recruitment of seedlings. Our results show a high proportion of clones and clonal size in sites with large areas of human-induced land use. Arnaud-Haond et al. (2010) found a correlation between mortality and clonal richness in *Posidonia* meadows with resilient sites having a higher number of large-sized clones due to successful competition under disturbance. *E. acoroides* has a very low rhizome growth rate (ca. 3 cm/year) (Marbà and Duarte, 1998) which suggests that large genets for this species may have persisted and weathered environmental change over hundreds of years. The selection of resistant genets under strong disturbance and the subsequent reproduction by clonal spread of these persistent genets may thus explain the positive effect of human-induced land use on clonality. Clonal growth and structure also have direct fitness assets including competitive advantages for space, higher reproductive success when multiple ramets have the potential to mate (Pan and Price, 2001) and the translocation of resources between ramets (Marbà et al., 2002; Jahnke et al., 2015) though no evidence for carbon allocation in turbid waters has been found for *E. acoroides* (Kiswara et al., 2005). Recent studies on *Z. marina* show that somatic mutations and epigenetic variation originated from asexual reproduction can create phenotypic variation between ramets on which selection can act and may result in the adaptation of genets toward environmental change (Jueterbock et al., 2020; Yu et al., 2020). Decreased levels of successful sexual reproduction may be an additional explanation for the negative effect of disturbance on clonal richness. Light availability has been identified as an important driver for flowering in *E. acoroides* with a lower proportion of female flowers and fruits in deeper and turbid sites due to decreased levels of photosynthesis (Rollón et al., 2003). Fragmentation of populations and reductions in shoot density which decreases pollen trapping potential of leaves and flowers can affect pollination and thus also fruit set (Vermaat et al., 2004). A negative association between clonal and sexual reproduction due to resource availability and allocation in individuals may exist but no evidence for this trade-off is currently present for *E. acoroides* (Jahnke et al., 2015).

### Ecological Significance and Conservation

Our findings highlight the importance of both sexual and asexual reproduction for the persistence of populations of *E. acoroides* in areas with large-scale human-induced land use. Meadows are sustained by clonal growth and local recruitment of seedlings with lagoons acting as barriers to gene flow. Large persistent genets contribute to the resilience of meadows of *E. acoroides* due to their resistance toward environmental pressures. However, recruitment by seedlings remains crucial for the colonization and recovery of distant populations, the maintenance of genetic diversity and the adaptation toward environmental change and should be prioritized in management though restricted connectivity among lagoons and local adaptation may complicate conservation measures. To fully understand the response and resilience of populations of *E. acoroides* toward disturbance, spatial analyses combined with temporal dynamics of clonal richness, structure and genetic diversity should be investigated in future research. Moreover, it is important to identify the direct local environmental drivers caused by human-induced land use and their relative effect on populations of *E. acoroides*. The studied meadows are most likely remnants of large, connected multi-species seagrass beds which disappeared with increasing human activities (Chen et al., 2016; Khanh Ni et al., 2020) and *E. acoroides* being the only remaining seagrass species persisting under high levels of human-induced disturbance. We hypothesize that seagrass ecosystems with *E. acoroides* as key species will eventually shift to systems dominated by macro-algae, epiphytic algae and phytoplankton when environmental conditions continue to diminish, combined with the effects of climate change and potential large disturbance events in the nearby future. Crucial steps for protection and recovery is to improve water quality by managing human-induced land use and related activities and maximizing ecosystem resilience taking into account all key actors (e.g., biological features of seagrass species and communities, functional important species trophic interactions, connectivity with other ecosystems) (Unsworth et al., 2015). Only then will it be effective to invest in practical management measures (e.g., planting seeds or the translocation of plant fragments) for the conservation of seagrass ecosystems.

## Data Availability Statement

The dataset containing microsatellite allele scores generated for this study is included within Data Sheet 1 of the Supplementary Material, further inquiries can be directed to the corresponding author.

## Author Contributions

JD: conceptualization (lead), investigation (lead), methodology (lead), collecting field data (lead), data curation (lead), formal analysis (lead), visualization (lead), writing-original draft (lead), writing-review and editing (lead), funding acquisition (supporting), and resources (supporting). TP: investigation (supporting), methodology (supporting), and collecting field data (supporting). QL: investigation (supporting), methodology (supporting), and collecting field data (supporting). LT: conceptualization (lead), investigation (supporting), methodology (supporting), collecting field data (supporting), data curation (supporting), formal analysis (supporting), visualization (supporting), writing-original draft (supporting), writing-review and editing (supporting), funding acquisition (lead), and resources (lead). All authors contributed to the article and approved the submitted version.

## Conflict of Interest

The authors declare that the research was conducted in the absence of any commercial or financial relationships that could be construed as a potential conflict of interest.

## Footnotes

1 https://scihub.copernicus.eu
