# Supplementary material for: Persistent Clones and Local Seed Recruitment Contribute to the Resilience of Enhalus acoroides Populations Under Disturbance
Source: Front Plant Sci. 2021 Jun 4;12:658213. doi: 10.3389/fpls.2021.658213 (PMC8248806; doi:10.3389/fpls.2021.658213)

Supplementary Figure 1. Spatial autocorrelation graphs on transect level for populations of *Enhalus acoroides* including the among ramets and among genets analyses over eight distance classes (0-1m, 1-2m, 2-3m, 3-5m, 5-10m, 10-20m, 20-30m, 30-50m) with the last distance class (30-50m) not graphically represented. 95% confidence intervals of kinship values (F_{ij}) are represented by dashed lines.

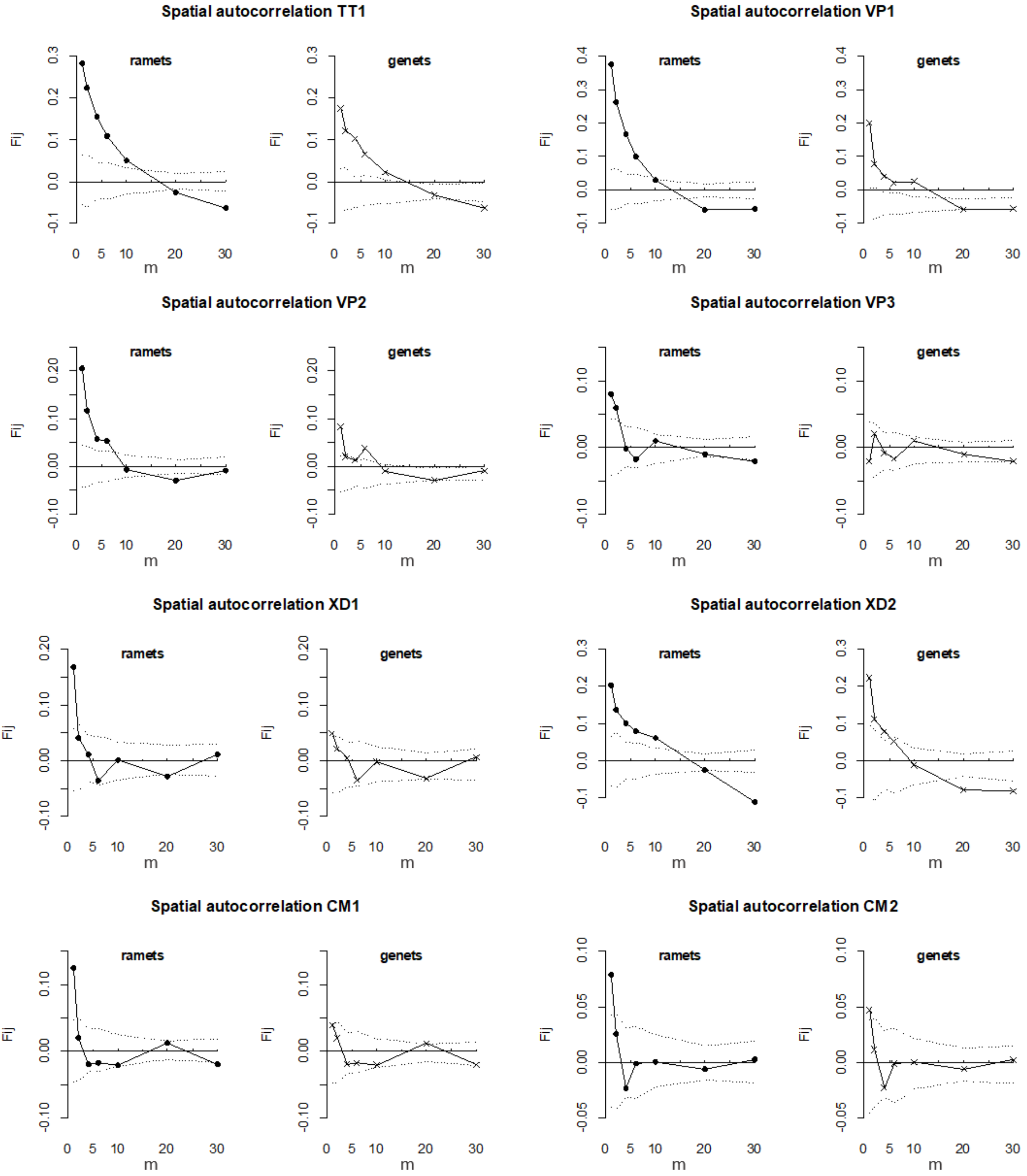

Supplement: Supplementary file 1 [file Image_1.PDF]
