# Supplementary material for: Persistent Clones and Local Seed Recruitment Contribute to the Resilience of Enhalus acoroides Populations Under Disturbance
Source: Front Plant Sci. 2021 Jun 4;12:658213. doi: 10.3389/fpls.2021.658213 (PMC8248806; doi:10.3389/fpls.2021.658213)

Supplementary Figure 2. Mean LnP(K) and Delta K for each number of groups (K) showing K=3 as the most likely based on Delta K (Evanno et al., 2005)

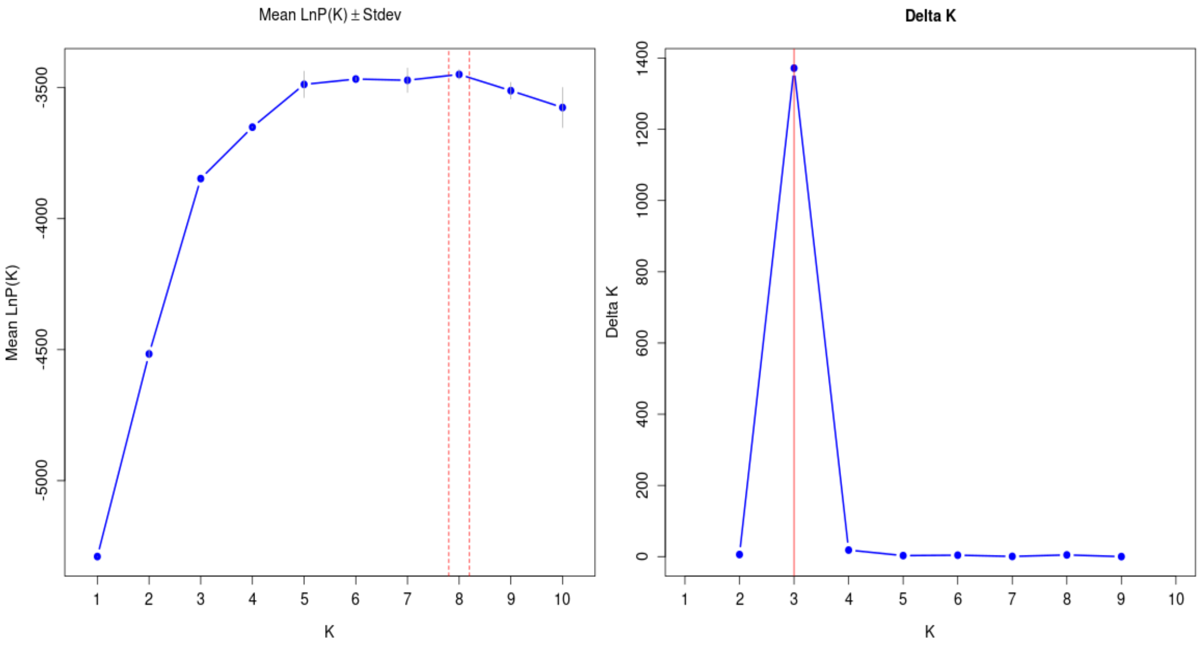

Supplement: Supplementary file 2 [file Image_2.PDF]
