# Supplementary material for: Persistent Clones and Local Seed Recruitment Contribute to the Resilience of Enhalus acoroides Populations Under Disturbance
Source: Front Plant Sci. 2021 Jun 4;12:658213. doi: 10.3389/fpls.2021.658213 (PMC8248806; doi:10.3389/fpls.2021.658213)

Supplementary Figure 3. Land use classification map of the four sampled lagoons. Borders of classification represent the delineation of the catchment area. Map generated using the Quantum Geographical Information System, version 3.10.3 (QGIS). Basemap source: ArcGIS.

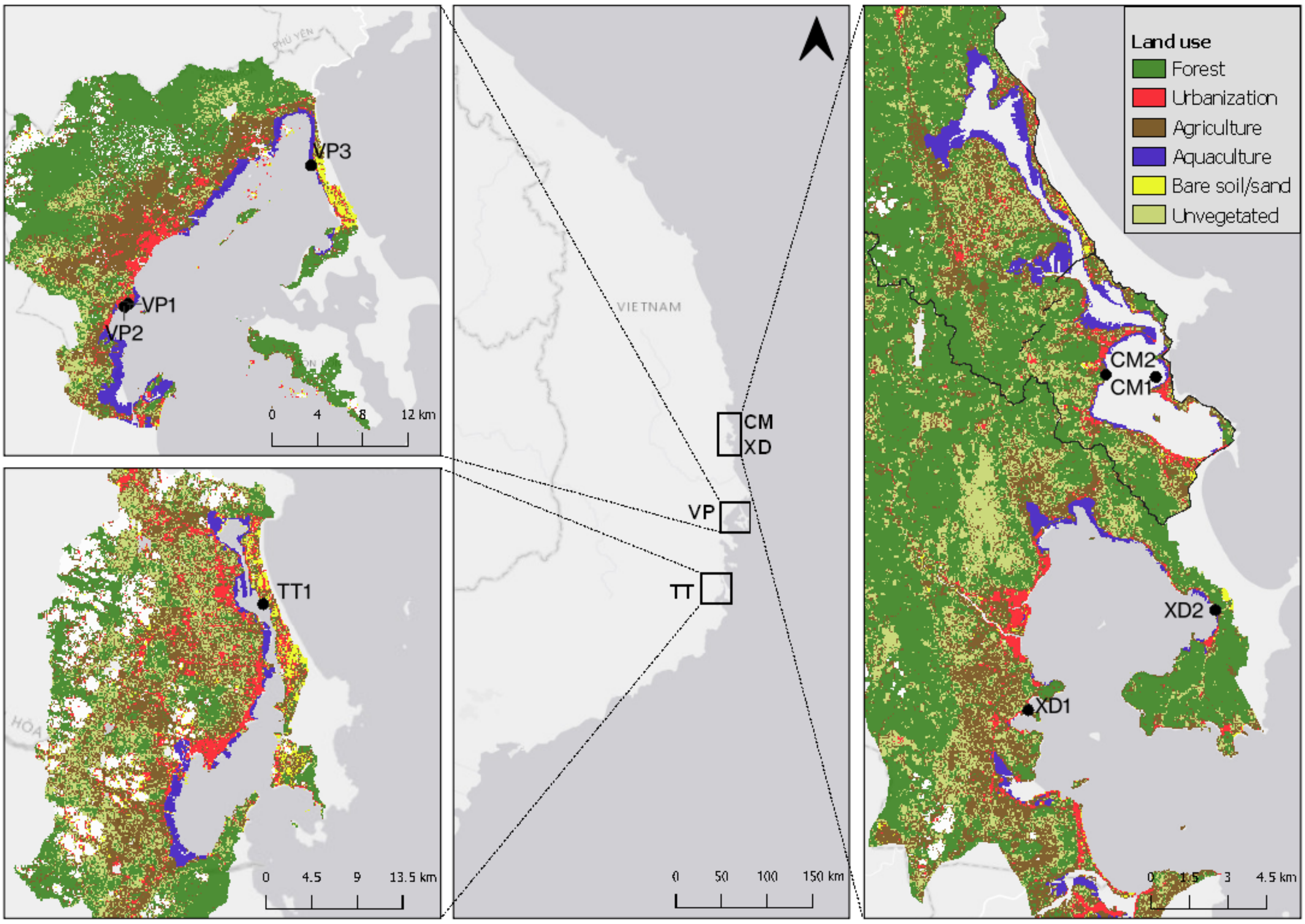

Supplement: Supplementary file 3 [file Image_3.PDF]
