# Supplementary material for: Persistent Clones and Local Seed Recruitment Contribute to the Resilience of Enhalus acoroides Populations Under Disturbance
Source: Front Plant Sci. 2021 Jun 4;12:658213. doi: 10.3389/fpls.2021.658213 (PMC8248806; doi:10.3389/fpls.2021.658213)
Supplement: Supplementary file 4 [file Table_1.DOCX]

**Supplementary Table 1.** Details of 11 primers and microsatellite loci for *Enhalus acoroides*. Total number of alleles (A), overall observed heterozygosity (H_obs_), overall expected heterozygosity (H_exp_).

| **Locus** | **Primer sequence (5'-3')** | **Repeat motive** | **Observed range** | **A** | **H_obs_** | **H_exp_** |
| --- | --- | --- | --- | --- | --- | --- |
| Ea1461 | F: < FAM > GTCTTCACCTAGATCCACATG | (AC) 13 | 276-280 | 3 | 0.394 | 0.342 |
|  | R: GCTAAACTTTGACTAACTCCC |  |  |  |  |  |
| Ea447 | F: < HEX > GGGGAGCTATCTTTGTGC | (TG) 14 (GA) 13 (TG) 5 | 205-211 | 4 | 0.443 | 0.443 |
|  | R: GAATCACCCGTTCCAAGA |  |  |  |  |  |
| Eaco_001 | F: GGCTTGAGTTTGTTTAGAATTCTAG | (TG)16 | 248-254 | 4 | 0.232 | 0.212 |
|  | R: TTACATGTGGAATGCATACAC |  |  |  |  |  |
| Eaco_002 | F: ACTTCAGCCTTGTATGGAACT | (TG)5CG(TG)4TATGTCTGTA(TG)8N28(TG)9 | 194-200 | 4 | 0.112 | 0.112 |
|  | R: CTATATGCCATAACCATCCATG |  |  |  |  |  |
| Eaco_009 | F: CAATCGTCCAATCCAAAGGC | (TG)13 | 148-154 | 3 | 0.391 | 0.371 |
|  | R: GGAGAATTGTATTATTTAC |  |  |  |  |  |
| Eaco_048 | F: CATAAGATGCGTGATCAAGC | (GT)8 | 164-166 | 2 | 0.013 | 0.012 |
|  | R: CATGCTACAACTGATCTTCTG |  |  |  |  |  |
| Eaco_050 | F: GAATAAATCAAGTCCCTTGAG | (TG)9TA(TG)5TATG(TA)8 | 250-258 | 5 | 0.312 | 0.322 |
|  | R: CAAATAAGATGTGGCTTAC |  |  |  |  |  |
| Eaco_051 | F: CATACAGATGCATGCATACTC | (GA)15GTGC(GT)16GC(GT)4 | 219-237 | 11 | 0.657 | 0.614 |
|  | R: CTAAGCGCTACGTGGTACTAG |  |  |  |  |  |
| Eaco_052 | F: CAGGCGCACAACGTATGTAC | (TG)4TC(TG)4TC(TG)5 | 142-153 | 2 | 0.020 | 0.028 |
|  | R: GAACCACATCATCAGTGTG |  |  |  |  |  |
| Eaco_054 | F: GCTTCTAATTAGCATTTTGGACTTCAG | (CT)15 | 278-307 | 13 | 0.627 | 0.620 |
|  | R: ATTTGGGACGTCCAAAGAG |  |  |  |  |  |
| Eaco_055 | F: CTTTTGCTCCCAAATTGAATG | (TC)18CG(TG)16 | 176-200 | 13 | 0.653 | 0.603 |
|  | R: ATGCTTAGTGCAGCTTGTTC |  |  |  |  |  |
