# Supplementary material for: Persistent Clones and Local Seed Recruitment Contribute to the Resilience of Enhalus acoroides Populations Under Disturbance
Source: Front Plant Sci. 2021 Jun 4;12:658213. doi: 10.3389/fpls.2021.658213 (PMC8248806; doi:10.3389/fpls.2021.658213)
Supplement: Supplementary file 5 [file Table_2.DOCX]

**Supplementary Table 2.** Results of fine-scale spatial autocorrelation analyses of *Enhalus acoroides* for eight populations along the South Central coast of Vietnam over eight distance classes (0-1m, 1-2m, 2-3m, 3-5m, 5-10m, 10-20m, 20-30m, 30-50m); kinship coefficient over first distance class (F_i1_), slope of regression (b) and *Sp-*statistic (S_p_) for kinship analyses between all pairs of ramets (ram) and between pairs of ramets of different genets (gen). Significance levels are indicated as follows: *** significant at p < 0.001, ** significant at p < 0.01, * significant at p < 0.05.

| **Site** | **F_i1ram_** | **b_ram_** | **Sp_ram_** | **F_i1gen_** | **b_gen_** | **Sp_gen_** |
| --- | --- | --- | --- | --- | --- | --- |
| TT1 | 0.283*** | -0.108*** | 0.151 | 0.176*** | -0.080*** | 0.097 |
| VP1 | 0.376*** | -0.132*** | 0.211 | 0.200*** | -0.078*** | 0.098 |
| VP2 | 0.205*** | -0.055*** | 0.070 | 0.084*** | -0.028*** | 0.03 |
| VP3 | 0.080** | -0.013** | 0.014 | -0.020 | 0.00 | 0.00 |
| XD1 | 0.168*** | -0.020*** | 0.024 | 0.050** | -0.005 | 0.005 |
| XD2 | 0.203*** | -0.095*** | 0.119 | 0.152*** | -0.081*** | 0.096 |
| CM1 | 0.125*** | -0.012* | 0.013 | 0.039* | -0.001 | 0.001 |
| CM2 | 0.079*** | -0.006 | 0.007 | 0.047* | -0.002 | 0.002 |
| Mean | 0.190 | -0.055 | 0.076 | 0.091 | -0.034 | 0.041 |
