# Supplementary material for: Persistent Clones and Local Seed Recruitment Contribute to the Resilience of Enhalus acoroides Populations Under Disturbance
Source: Front Plant Sci. 2021 Jun 4;12:658213. doi: 10.3389/fpls.2021.658213 (PMC8248806; doi:10.3389/fpls.2021.658213)
Supplement: Supplementary file 6 [file Table_3.docx]

**Supplementary Table 3.** Pairwise population F_ST_ estimates (below diagonal) and p-values (above diagonal).

|  | **TT1** | **VP1** | **VP2** | **VP3** | **XD1** | **XD2** | **CM1** | **CM2** |
| --- | --- | --- | --- | --- | --- | --- | --- | --- |
| **TT1** |  | 0.001 | 0.001 | 0.001 | 0.001 | 0.001 | 0.001 | 0.001 |
| **VP1** | 0.233 |  | 0.020 | 0.001 | 0.001 | 0.001 | 0.001 | 0.001 |
| **VP2** | 0.268 | 0.022 |  | 0.001 | 0.001 | 0.001 | 0.001 | 0.001 |
| **VP3** | 0.210 | 0.127 | 0.130 |  | 0.001 | 0.001 | 0.001 | 0.001 |
| **XD1** | 0.358 | 0.503 | 0.506 | 0.479 |  | 0.001 | 0.001 | 0.001 |
| **XD2** | 0.273 | 0.430 | 0.441 | 0.413 | 0.085 |  | 0.001 | 0.001 |
| **CM1** | 0.358 | 0.271 | 0.277 | 0.339 | 0.518 | 0.474 |  | 0.001 |
| **CM2** | 0.358 | 0.264 | 0.282 | 0.341 | 0.496 | 0.459 | 0.038 |  |
