# Supplementary material for: Persistent Clones and Local Seed Recruitment Contribute to the Resilience of Enhalus acoroides Populations Under Disturbance
Source: Front Plant Sci. 2021 Jun 4;12:658213. doi: 10.3389/fpls.2021.658213 (PMC8248806; doi:10.3389/fpls.2021.658213)
Supplement: Supplementary file 7 [file Table_4.DOCX]

**Supplementary Table 4.** Overall accuracy, Kappa accuracy, User’s accuracy and producer’s accuracy for six land use classes.

|  | **Overall accuracy (%)** | **Kappa  accuracy (%)** |
| --- | --- | --- |
| Total | 90.48 | 87.74 |
|  | **User’s accuracy (%)** | **Producer’s accuracy (%)** |
| Forest | 92.43 | 92.63 |
| Water | 99.95 | 98.61 |
| Urbanization | 96.33 | 97.05 |
| Agriculture | 89.87 | 89.86 |
| Bare soil | 98.08 | 96.96 |
| Unvegetated | 71.72 | 72.18 |
