# Supplementary material for: Persistent Clones and Local Seed Recruitment Contribute to the Resilience of Enhalus acoroides Populations Under Disturbance
Source: Front Plant Sci. 2021 Jun 4;12:658213. doi: 10.3389/fpls.2021.658213 (PMC8248806; doi:10.3389/fpls.2021.658213)
Supplement: Supplementary file 8 [file Table_5.DOCX]

**Supplementary Table 5.** Confusion matrix for six land use classes.

|  | **Reference class** | | | | | |  |
| --- | --- | --- | --- | --- | --- | --- | --- |
|  | **Forest** | **Water** | **Urban** | **Agri** | **Bare** | **Unveg** | **Sum** |
| **Forest** | 8508 | 0 | 0 | 253 | 0 | 444 | 9205 |
| **Water** | 0 | 4103 | 0 | 0 | 2 | 0 | 4105 |
| **Urban** | 0 | 5 | 3227 | 59 | 14 | 45 | 3350 |
| **Agri** | 175 | 48 | 81 | 8935 | 18 | 685 | 9942 |
| **Bare** | 0 | 5 | 0 | 11 | 1276 | 9 | 1301 |
| **Unveg** | 502 | 0 | 17 | 685 | 6 | 3069 | 4279 |
| **Sum** | 9185 | 4161 | 3325 | 9943 | 1316 | 4252 | 32182 |
