# Supplementary material for: Persistent Clones and Local Seed Recruitment Contribute to the Resilience of Enhalus acoroides Populations Under Disturbance
Source: Front Plant Sci. 2021 Jun 4;12:658213. doi: 10.3389/fpls.2021.658213 (PMC8248806; doi:10.3389/fpls.2021.658213)
Supplement: Supplementary file 9 [file Table_6.DOCX]

**Supplementary Table 6.** Area per land use class and catchment area for eight populations of *Enhalus acoroides* along the South Central coast of Vietnam for five buffers (0.5km, 1km, 5km, 10km, lagoon).

| **Buffer** | **Pop** | **Forest (km^2^)** | **Urban (km^2^)** | **Agr (km^2^)** | **Bare (km^2^)** | **Unveg**  **(km^2^)** | **Aqua**  **(km^2^)** | **Water**  **(km^2^)** | **Catch**  **(km^2^)** |
| --- | --- | --- | --- | --- | --- | --- | --- | --- | --- |
| 0.5km | TT1 | 0.01 | 0.03 | 0.13 | 0.10 | 0.01 | 0.00 | 0.49 | 0.28 |
|  | VP1 | 0.00 | 0.21 | 0.07 | 0.01 | 0.01 | 0.02 | 0.47 | 0.32 |
|  | VP2 | 0.00 | 0.17 | 0.02 | 0.00 | 0.00 | 0.02 | 0.58 | 0.21 |
|  | VP3 | 0.00 | 0.06 | 0.12 | 0.07 | 0.01 | 0.12 | 0.50 | 0.38 |
|  | XD1 | 0.08 | 0.05 | 0.13 | 0.01 | 0.08 | 0.00 | 0.42 | 0.35 |
|  | XD2 | 0.29 | 0.01 | 0.09 | 0.01 | 0.03 | 0.03 | 0.33 | 0.47 |
|  | CM1 | 0.13 | 0.05 | 0.12 | 0.00 | 0.03 | 0.04 | 0.39 | 0.38 |
|  | CM2 | 0.00 | 0.03 | 0.05 | 0.00 | 0.00 | 0.06 | 0.69 | 0.15 |
| 1km | TT1 | 0.04 | 0.24 | 0.51 | 0.31 | 0.08 | 0.02 | 1.87 | 1.20 |
|  | VP1 | 0.06 | 0.80 | 0.36 | 0.01 | 0.11 | 0.31 | 1.44 | 1.65 |
|  | VP2 | 0.01 | 0.71 | 0.37 | 0.01 | 0.09 | 0.23 | 1.67 | 1.42 |
|  | VP3 | 0.01 | 0.10 | 0.25 | 0.64 | 0.08 | 0.28 | 1.70 | 1.36 |
|  | XD1 | 0.43 | 0.15 | 0.80 | 0.02 | 0.32 | 0.01 | 1.36 | 1.73 |
|  | XD2 | 0.93 | 0.04 | 0.21 | 0.15 | 0.08 | 0.13 | 1.14 | 1.53 |
|  | CM1 | 0.44 | 0.24 | 0.36 | 0.05 | 0.21 | 0.12 | 1.66 | 1.42 |
|  | CM2 | 0.03 | 0.26 | 0.24 | 0.02 | 0.04 | 0.14 | 1.88 | 0.72 |
| 5km | TT1 | 3.06 | 9.31 | 13.87 | 4.43 | 6.88 | 5.29 | 8.75 | 42.83 |
|  | VP1 | 6.98 | 4.68 | 15.28 | 0.26 | 7.12 | 3.48 | 38.08 | 37.80 |
|  | VP2 | 7.40 | 4.44 | 15.73 | 0.25 | 7.72 | 3.73 | 35.62 | 39.28 |
|  | VP3 | 0.29 | 1.69 | 3.90 | 3.83 | 0.66 | 3.94 | 37.04 | 14.31 |
|  | XD1 | 14.35 | 3.01 | 16.76 | 0.24 | 9.17 | 0.70 | 32.75 | 44.23 |
|  | XD2 | 14.09 | 0.42 | 2.31 | 0.22 | 1.72 | 0.47 | 19.04 | 19.23 |
|  | CM1 | 15.35 | 1.92 | 7.32 | 0.34 | 6.14 | 3.11 | 14.70 | 34.19 |
|  | CM2 | 9.96 | 1.98 | 6.60 | 0.36 | 4.89 | 2.26 | 14.28 | 26.05 |
| 10km | TT1 | 16.64 | 27.14 | 60.22 | 10.83 | 40.15 | 11.02 | 22.34 | 166.01 |
|  | VP1 | 31.39 | 12.46 | 61.27 | 0.81 | 27.61 | 12.73 | 121.44 | 146.27 |
|  | VP2 | 31.28 | 12.63 | 60.27 | 0.85 | 27.64 | 12.71 | 117.52 | 145.38 |
|  | VP3 | 31.50 | 4.61 | 23.91 | 5.63 | 8.19 | 8.49 | 88.41 | 82.32 |
|  | XD1 | 67.17 | 5.91 | 41.06 | 0.73 | 29.73 | 3.45 | 84.96 | 148.05 |
|  | XD2 | 25.60 | 3.62 | 16.66 | 0.45 | 12.44 | 2.09 | 79.14 | 60.87 |
|  | CM1 | 30.73 | 2.97 | 18.11 | 0.72 | 13.57 | 5.18 | 16.94 | 71.27 |
|  | CM2 | 25.37 | 2.89 | 15.47 | 0.71 | 11.61 | 4.79 | 16.20 | 60.84 |
| Lagoon | TT1 | 205.76 | 62.17 | 253.41 | 24.34 | 162.91 | 25.18 | 139.24 | 733.78 |
|  | VP1 | 213.05 | 20.25 | 109.69 | 7.52 | 47.50 | 22.66 | 301.08 | 420.66 |
|  | VP2 | 213.05 | 20.25 | 109.69 | 7.52 | 47.50 | 22.66 | 301.08 | 420.66 |
|  | VP3 | 213.05 | 20.25 | 109.69 | 7.52 | 47.50 | 22.66 | 301.08 | 420.66 |
|  | XD1 | 147.56 | 6.25 | 49.81 | 0.74 | 40.82 | 3.45 | 86.91 | 248.63 |
|  | XD2 | 147.56 | 6.25 | 49.81 | 0.74 | 40.82 | 3.45 | 86.91 | 248.63 |
|  | CM1 | 86.40 | 3.36 | 24.34 | 0.83 | 19.51 | 8.44 | 20.55 | 142.89 |
|  | CM2 | 86.40 | 3.36 | 24.34 | 0.83 | 19.51 | 8.44 | 20.55 | 142.89 |
